# Impact of Cold Stress on Physiological, Endocrinological, Immunological, Metabolic, and Behavioral Changes of Beef Cattle at Different Stages of Growth

**DOI:** 10.3390/ani13061073

**Published:** 2023-03-16

**Authors:** Won-Seob Kim, Jalil Ghassemi Nejad, Hong-Gu Lee

**Affiliations:** Department of Animal Science and Technology, Sanghuh College of Life Sciences, Konkuk University, Seoul 05029, Republic of Korea

**Keywords:** cold stress, beef cattle, physiological parameters, blood parameters, behavior patterns

## Abstract

**Simple Summary:**

This study explored the effects of cold stress on the physiological, blood, and behavioral parameters of beef cattle according to the growth stage. We found that cold stress alters the heart rate, rectal temperature, blood cortisol, glucose, non-esterified fatty acids, and time spent standing at different stages of both beef calves and beef steers. Thus, the finding indicated that physiological, blood, and behavioral changes are potent biomarkers of cold stress in beef cattle.

**Abstract:**

The purpose of this study was to investigate the effect of cold stress (CS) on the physiological, blood, and behavioral parameters of beef cattle according to their growth stage. Twelve calves in the growing stages (220.4 ± 12.33 kg, male and non-castrated) and twelve steers in the early fattening stages (314.2 ± 18.44 kg) were used in this experiment. The animals were randomly distributed into three homogenized groups (four animals each) for 14 days, namely threshold, mild–moderate cold stress (MCS), and extreme cold stress (ECS), according to the outside ambient temperature. The feed and water intakes were recorded daily. The physiological parameters, blood parameters, and behavioral patterns were measured weekly. All data were analyzed using repeated-measures analysis. The calves exposed to the ECS decreased (*p* < 0.064, tendency) their dry matter intake compared to the threshold and MCS groups. The HR and RT increased (*p* < 0.001) in the ECS compared to the threshold in calves and steers. Moreover, increased (*p* < 0.05) blood cortisol, non-esterified fatty acids (NEFA), and time spent standing were observed after exposure to ECS in calves and steers. However, the calves exposed to the ECS had decreased (*p* = 0.018) blood glucose levels compared to the threshold. In conclusion, ECS affects the dry matter intake, HR, RT, blood cortisol, NEFA, and behavioral patterns in beef calves and steers. This phenomenon indicated that beef cattle exposed to CS modulated their behavior and blood parameters as well as their physiological response to maintain homeostasis regardless of the growth stage.

## 1. Introduction

In the near future, it is anticipated that climate change will increase, including extreme weather occurrences [1]. In South Korea, the mean minimum temperatures and relative humidity from December to February over the last 30 years are −3.2 to −5.5 °C and 56.2 to 65.5%, respectively [2]. Cattle experience cold stress (CS) during the winter season, and these low environmental temperatures affect their growth performance [3,4], metabolic status [3,5], and immune function [4,6]. CS in beef cattle reduces animal welfare while increasing economic losses for the industry [3,7,8,9].

Changes in animal growth performance are known to occur due to seasonal changes in environmental and climatic conditions [10]. These changes are likely due to differences in adaptation to essential energy demand and energy utilization efficiency [11]. Many previous studies have been conducted on beef cattle to determine how environmental conditions, such as ambient temperature (AT), affect animal performance [3,12]. Prolonged exposure to cold environments can lead to CS, which involves a variety of temperature regulation mechanisms to ensure that the maintenance requirements remain constant until the threshold temperatures are exceeded [7]. Metabolic acclimation caused by cold exposure was thought to reduce animal performance and efficiency compared to animals not exposed to such conditions at the same feed intake [8]. Previous research found that an increased metabolic rate causes an increased heart rate (HR), rectal temperature (RT), deep breathing, and muscle shivering in animals exposed to severe CS, as well as an increase in energy requirements [6,13]. However, these intake and physiological parameters do not reflect the response of the internal performance, especially the energy metabolism, hormones, molecular chaperones, and blood metabolites. In beef cattle, blood glucose and non-esterified fatty acid (NEFA) levels are highly influenced by feed intake due to changes in the energy metabolism in the body [12,14]. Moreover, blood cortisol, a stress hormone, is known to change in response to acute stress conditions and to regulate neural sympathy to correlate with physiological indicators such as HR and RT [14]. In response to cold temperatures, heat shock protein 70 (HSP70) has also been observed to accumulate [15,16]. A previous study reported that HSP70 gene expression was increased after being exposed to −32 °C CS for 3 h in cattle [6]. This molecular chaperone aids in maintaining homeostasis and protecting cells from damage. This molecular chaperone assists in preserving homeostasis and protecting cells from environmental damage [17].

Behavioral patterns, including standing and lying down time, are also important indicators after exposure to CS conditions [18,19], because cattle can maximize their effective surface area for heat dissipation from the body surface by changing their behavior. Kaygusuz and Akdağ [18] reported increased standing positions and decreased lying positions when Simmental cows were exposed to CS. This phenomenon indicated that wet barn floors influenced cattle behavior, and they preferred to stand rather than lie to balance their body heat.

Different growth stages in cattle respond differently to energy requirements and metabolic responses, resulting in different resistance to CS. There is, however, little information on how CS affects the physiological parameters, blood hormones, molecular chaperones, metabolites, and behavioral patterns of beef cattle according to the growth stage. We hypothesized that different responses to the stress parameters of CS would appear differently depending on the growth stage of the beef cattle. Therefore, the objective of the study was to evaluate the various stress parameters, such as the physiological, blood, and behavioral responses after exposure to CS conditions in Korean native beef calves in the growing stage (6–7 months old) and Korean native steers in the early fattening stages (12–13 months old).

## 2. Materials and Methods

All procedures involving animals were approved by the Institutional Animal Care and Use Committee (IACUC) of Konkuk University (Approval No: KU15201).

### 2.1. Animals, Experimental Design, and Diets

The study was conducted at the Konkuk University experimental farm (Chungju, Chungcheongbuk-do, Republic of Korea), located at 36°58′ north latitude and 127°56′ east longitude. Twelve Korean native calves in the growing stage (209.9 ± 10.90 days old with body weight (BW) of 220.4 ± 12.33 kg, male and non-castrated) and twelve Korean native steers in the early fattening stages (324.3 ± 15.86 days old with BW of 314.2 ± 18.44 kg) were used in this experiment. The animals were randomly distributed into three homogenized groups (four calves and four steers each), and each group was exposed to CS under a natural environment, namely threshold, mild–moderate cold stress (MCS), and extreme cold stress (ECS) according to the outside AT. The total experiment lasted 14 days for each group. All of the animals were kept in individual pens during the entire period to calculate the feed and water intake. The animals were subjected to an acclimatization period of seven days in individual pens for adaptation using an individual feed and water approach system. Four sensors were used during the experiment to record the AT and relative humidity (RH) at 1-s intervals (SHT7x, Sensirion AG, Laubisruetistrasse 508712, Staefa ZH, Switzerland). The experimental farm was covered with a roof, and the animals were raised indoors; therefore, they were protected from rainfall, wind, and direct sunlight during the experiment. Only the AT and RH affected the CS conditions during the total period.

The diets used in this experiment were composed of 40% roughage (Timothy, *Phleum pratense* L.) and 60% concentrate. The chemical compositions of the feed are shown in Table 1. At 0900 and 1700 h, the feed was weighted and offered twice a day. At 0900, 1300, 1700, and 2100 h, water was weighted and offered four times per day. The next day (0900 h) after the offering, the residues of both feed and water were noted on a daily basis.

### 2.2. Chemical Analysis of Diets

According to AOAC [20], the following measurements were analyzed: dry matter (DM; method 930.15), crude protein (CP; method 984.13), acid detergent fiber (ADF; method 973.18), ether extract (EE; method 920.39), and ash (method 942.05). The method used by Van Soest et al. [21,22] to analyze the neutral detergent fiber (NDF) and acid detergent lignin (ADL) content. Coupled plasma spectroscopy (method 945.46) was used to inductively estimate the Ca and P levels [20]. Ash content was determined by incineration at 550 °C overnight in a muffle furnace, and dry matter was assessed by drying ground diets in a vacuum oven at 100 °C overnight (KMF-500, Lab Corporation, Seoul, Republic of Korea). The Kjeltec™ System (Kjeltec™ 2400, FOSS, Hillerod, Denmark) was used to assess the total nitrogen in the feed to determine the CP contents. The final CP content was calculated as nitrogen × 6.25. The ether extraction system (ANKOMXT15 Extractor, ANKOM Technology, Macedon, NY, USA) was used to measure the amount of ether extracted.

### 2.3. Physiological Parameters under Cold Stress

The physiological parameters, including the HR and RT, were measured every week at 1400 h. A large clinical animal thermometer (TES-1300 Thermometer; E&E PROCESS Instrument Co., Vaughan, ON, Canada) was used to measure the rectal temperature. It was put into the rectum of beef steers to a depth of 3 cm and kept in contact with the mucosa for 1 min. A stethoscope (TS-DIA01002; Tenso Medical Instrument Co., Ningbo, China) was put directly onto the left thoracic region under one of the auscultation foci for 1 min in order to measure heart rate, which is expressed in beats per minute (BPM).

### 2.4. Blood and Behavior Parameters under Cold Stress

Every week at 1100 h, blood samples from the jugular veins of beef steers were taken for serum extraction (20 mL; Becton-Dickinson, Belliver Industrial Estate, PL6 7BP, Plymouth, UK) and hematology (4 mL; Becton-Dickinson, Franklin Lakes, NJ, USA) analysis. Serum samples were obtained from blood samples after centrifugation at 2700× *g* for 15 min at 4 °C. Serum was transferred to a 1.5 mL tube (Eppendorf AG, Hamburg, Germany) and kept at −80 °C until analysis.

Using a commercial bovine ELISA test kit, the levels of serum cortisol and HSP70 were examined (Life Diagnostics, Inc., West Chester, PA, USA; Endocrine Technologies, Inc., Newark, CA, USA). JW Medical (Seoul, Republic of Korea) provided the analytical reagents to measure the levels of glucose. Wako Pure Chemical (Osaka, Japan) provided the analytical reagents to measure the levels of non-esterified fatty acids (NEFA). An automated chemical analyzer (Hitachi 7180, Tokyo, Japan) was used to measure all metabolites. Using a VetScan HM2 (Diamond Diagnostics, Abaxix Inc., Holliston, MA, USA), whole blood was analyzed for white blood cell (WBC) and platelet counts as hematological traits.

Four cameras (SNV-7080R, Hanwha Techwin, Changwon, Republic of Korea) were used to record standing and lying down behavioral patterns. Every week, between the hours of 0900 and 1900, the times spent standing or lying down were recorded (600 min).

### 2.5. Statistical Analysis

The repeated-measures analysis and the GLM procedure in SAS version 9.4 (SAS Institute Inc., Cary, NC, USA) were used to examine all the data. The model used was as follows:

Y*_ijk_* = µ + α*_i_* + β*_j_* + γ(α)*_ik_* + ε*_ijk_*

where Y*_ijk_* was the observation of beef steer *k* at sampling time *j* for a given treatment *i*, µ was the overall mean, α*_i_* was the fixed effect of treatment *i* (threshold, MCS, and ECS), β*_j_* was the fixed effect of sampling time *j* (every week), γ(α)*_ik_* was the random effect of animal *k* nested in treatment *i*, and ε*_ijk_* was the residual effect. The model included a random effect for animals’ identification. The subject of the REPEATED statement was the effect of beef steers. For mean comparisons, Tukey’s honest significant difference (HSD) test was used. The covariance structures (autoregressive order 1, unstructured, and compound symmetry) for the repeated measures model were tested. The structure that best fits the model was chosen based on the smallest value of Schwarz’s Bayesian information criterion. The first day of sampling in each group was included as a covariate to adjust the means. The covariate factor was included in the model when appropriate but was removed from the model when it was insignificant.

To confirm the analysis of the difference between groups in this study, we performed a post hoc power analysis using G × Power (version 3.1.9.7, University of Dusseldorf, Dusseldorf, Germany). The post hoc power analysis was applied with α = 0.05, sample size = 12, and an effect size = 0.71 for beef calves and an effect size = 0.75 for beef steers. The power of analysis (1 − β) for the difference among the groups was 0.81 for beef calves and 0.85 for beef steers. The least square means of the data are presented, along with standard errors. Differences were considered statistically significant if the *p*-value was less than 0.05. That is, *p*-values between 0.05 and 0.10 reflected a tendency to differ significantly.

## 3. Results

### 3.1. Ambient Temperatures and Relative Humidity during the Experiment

The mean, maximum, and minimum of the AT and RH were lower in the ECS period compared to the threshold and MCS periods (Table 2).

### 3.2. Intake and Physiological Parameters

The average dry matter intake and water intake were calculated every week for the entire experiment. The calves exposed to the ECS had a decreased (*p* = 0.064, tendency) dry matter intake compared to the threshold and the MCS (Table 3). However, the dry matter intake did not differ (*p* = 0.147) significantly among the groups of steers (Table 3). The water intake was not different (*p* > 0.10) among the groups of both calves and steers (Table 3).

The physiological parameters, including the HR and RT, were measured every week. The HR and RT increased (*p* < 0.001) in the ECS compared to the threshold in the calves and steers (Table 4).

### 3.3. Blood Parameters

The blood cortisol, metabolites, and hematological parameters were measured every week during the total period of the experiment. The calves and steers exposed to the ECS significantly increased (*p* < 0.05) their blood cortisol and NEFA levels compared to the threshold (Table 5). Decreased (*p* = 0.018) blood glucose levels were observed after exposure to ECS compared to the threshold in calves. However, the blood glucose levels did not differ (*p* = 0.257) significantly among the steer groups (Table 5). The heat shock protein 70 and hematological parameters, including the WBC, lymphocytes, and platelets, were not different (*p* > 0.10) among the groups of calves and steers (Table 5).

### 3.4. Behavioral Parameters

The times spent in standing and lying down positions were calculated every week from 0900 to 1900 h (600 min/day). The calves (*p* < 0.001) and steers (*p* = 0.022) exposed to ECS significantly increased their time spent standing compared to the threshold (Table 6). In contrast, time spent lying down was decreased in the ECS for the calves (*p* < 0.001) and steers (*p* = 0.022) (Table 6).

## 4. Discussion

A variety of stressors have an impact on livestock growth performance, production, metabolic disturbances, immune function, and animal welfare. A cold environment is one of the major factors affecting productivity and economic losses in the beef cattle industry [3,7]. According to the Intergovernmental Panel on Climate Change (IPCC), extreme weather events in the winter are likely to become more frequent in the near future [1]. Cold stress causes physiological, metabolic, and immune status changes in cattle to maintain homeostasis, resulting in lower productivity [3,18,19]. Previous studies have reported that after CS exposure, cattle physiological responses such as the respiration rate, HR, and RT change to maintain homeostasis [7,19]. However, the internal performance, particularly the energy metabolism, hormones, and blood metabolites, is not reflected by these parameters. Moreover, as beef cattle grow and their metabolic mechanisms and cold resistance change, it can result in other CS responses in beef cattle according to the growth stage [3,19,23]. Consequently, the results of this study could help us better understand how CS can compromise beef cattle capacity.

CS is generally affected not only by AT and RH but also by other environmental factors such as air velocity and wind speed [18]. However, beef cattle grown in indoor feedlots are protected from rain, wind, and direct sunlight during winter because the farm is covered by roofs and curtains. Therefore, in this study, we assumed that only AT and RH influenced CS in animals. Several studies have already defined the temperature–humidity index, which divides thermal stress ranges in cattle [12,24], but the CS range has not yet been defined. In the current study, the minimum, maximum, and mean AT and RH were significantly different between the experiment periods. Thus, the minimum AT and RH during the second (−8.23 °C, 64.32%) and third (−2.37 °C, 44.74%) periods could be associated with mild–moderate or extreme CS, whereas the first period (0.60 °C, 62.24%) was considered the threshold for Korean native beef cattle.

Previous studies have reported that when animals were exposed to CS during the winter season, they required more energy to maintain homeostasis via heat production [25,26]. Mujibi et al. [11] reported increased dry matter intake, residual feed intake, and feeding behavior with exposure to CS in young, growing beef cattle. However, in contrast, another study found no difference in the growth performance (average daily gain and gain-to-feed ratio) during the winter season in Korean native steers [23]. Given these results [11,23], the resistance to CS differs depending on the breed, age, and feeding system. In the current study, the dry matter intake showed different trends between calves in the growing stage and steers in the early fattening stage. The dry matter intake was reduced in beef calves during exposure to low temperature environmental conditions. This reduction led to decreased blood glucose levels during CS in calves in the growing stage (6–7 months old). However, the blood NEFA levels were increased after exposure to ECS. This phenomenon suggested that growing calves exposed to CS used stored protein and fat as energy sources to maintain body–heat–producing mechanisms rather than lowering their nutrient intake and blood glucose levels. Moreover, the blood NEFA levels increased after exposure to ECS in steers in the early fattening stage without changes in the dry matter intake or blood glucose levels in this study. These findings suggested that beef steers (12–13 months old) may use both glucose and other stored nutrients as energy sources to maintain skeletal muscle growth during the winter season.

The physiological parameters, including HR and RT, and the blood cortisol level are the most widely used indicators when cattle are exposed to extreme changes in external environmental conditions. Under CS conditions, the HR and RT responded to homeostatic disturbances, resulting in a range of physiological changes. In the current study, the ECS period groups of both calves and steers had increased HR and RT. Previous research found that animals exposed to acute CS for the first time without acclimation had an increased HR and RT, which is consistent with the stress response in this study [27,28]. The HR and RT can be used as indicators of thermal balance in the body to assess the adverse effects of environmental stress on growth performance and productivity [12,14,29]. The cortisol levels in the blood are associated with abnormal animal behaviors such as anxiety and sensitivity [30]. Sejian et al. [31] reported that the hypothalamic–pituitary–adrenal and the sympathetic–adrenal medullary axes can maintain homeostasis in response to environmental change by stimulating the blood cortisol levels in animals. The primary function of blood cortisol is to maintain the body’s homeostasis by stimulating the immune system during times of acute stress [32]. In the current study, the blood cortisol levels were increased after exposure to ECS in both calves and steers. This phenomenon acted to maintain homeostasis during the winter season regardless of the beef cattle’s growth stage. The HSP70 is one of the chaperones closely associated with CS [5,33]. A previous study reported that short-term exposure to CS increased HSP70 levels in beef cattle [6]. In addition, another study showed that HSP70 was immediately increased after cold shock in the immune cells of Sahiwal and Frieswal cattle [34]. However, blood HSP70 levels did not change in both calves and steers in this current study. This phenomenon is similar to the results of our previous study [12]. It might be considered that the molecular chaperone, which increased rapidly in the early period after exposure to CS, reached homeostasis with the passage of time and recovered. Taken together, the HR, RT, and blood cortisol levels were closely associated with CS and may be used as sensitive indicators for determining the physiological stress levels in both beef calves in the growing stage and beef steers in the early fattening stage.

During the cold environmental conditions, the behavioral patterns, including time spent standing and lying down, changed in both beef calves and steers in this study. The lower temperature during winter causes there to be less dry bedding in the feedlot, which increases the moisture on the cattle’s skin and reduces the perceived temperature. A previous study also found that lying behavior decreased during extreme cold weather in winter while standing increased [18]. More standing time may allow for more surface area to be used to reduce skin moisture in beef cattle during the winter season.

## 5. Conclusions

This study explored the effects of cold stress on the physiological, blood, and behavioral parameters of beef cattle according to the growth stage. Overall, the heart rate, rectal temperature, blood cortisol, and behavioral patterns (standing and lying down) appeared to have similar patterns in both calves and steers except for the dry matter intake and blood glucose levels during the winter season. These parameters were closely associated with cold stress and may be used as sensitive indicators for determining stress levels. The results of the present study further improve our understanding of the responses of beef cattle to exposure to cold stress according to the growth stage.

## Figures and Tables

**Table 1:** Chemical compositions of experimental diets provided to the Korean native calves in the growing stage and the Korean native steers in the early fattening stage.

Chemical Composition ^1^	Concentrates (Calves)	Concentrates (Steers)	Roughage (Timothy Grass)
Composition, % of DM			
CP	18.19	14.50	5.81
NDF	30.63	26.23	67.38
ADF	12.08	8.98	39.03
ADL	4.31	3.82	5.23
EE	3.66	3.00	0.74
Crude Ash	7.41	12.00	5.01
Ca	1.05	1.10	0.10
P	0.46	0.50	0.19

^1^ DM = dry matter, CP = crude protein, NDF = neutral detergent fiber, ADF = acid detergent fiber, ADL = acid detergent lignin, EE = ether extract, Ca = calcium, P = phosphorus.

**Table 2:** The change in the ambient temperature and relative humidity during the experimental period.

Items ^1^	Threshold	MCS ^2^	ECS ^3^	SEM	*p*-Value
AT (°C)					
Mean	4.66 ^a^	−1.05 ^b^	−4.33 ^c^	0.733	<0.001
Maximum	12.25 ^a^	6.74 ^b^	2.56 ^c^	1.030	<0.001
Minimum	0.60 ^a^	−2.37 ^b^	−8.23 ^c^	0.857	<0.001
RH (%)	62.24 ^a^	64.32 ^a^	44.74 ^b^	3.316	<0.001

^1^ AT = ambient temperature, RH = relative humidity. ^2^ MCS = mild–moderate cold stress. ^3^ ECS = extreme cold stress. ^a–c^ Mean values with different letters differ significantly (*p* < 0.05).

**Table 3:** The effect of cold stress on the dry matter intake and water intake of Korean native calves in the growing stage and Korean native steers in the early fattening stage.

Items ^1^	Threshold	MCS ^1^	ECS ^2^	SEM	*p*-Value
Dry matter intake (kg/day)					
calves	4.44	4.38	4.02	0.127	0.064
steers	8.04	7.98	7.68	0.135	0.147
Water intake (L/day)					
calves	29.84	27.48	28.21	1.130	0.337
steers	46.50	46.25	44.88	1.104	0.544

^1^ MCS = mild–moderate cold stress. ^2^ ECS = extreme cold stress.

**Table 4:** The effect of cold stress on the heart rate and rectal temperature of Korean native calves in the growing stage and Korean native steers in the early fattening stage.

Items ^1^	Threshold	MCS ^2^	ECS ^3^	SEM	*p*-Value
HR (bpm)					
calves	61.63 ^b^	65.38 ^b^	71.38 ^a^	1.447	<0.001
steers	62.75 ^b^	73.63 ^a^	74.88 ^a^	0.977	<0.001
RT (°C)					
calves	38.98 ^b^	39.24 ^b^	39.63 ^a^	0.097	<0.001
steers	38.61 ^c^	39.08 ^b^	39.34 ^a^	0.042	<0.001

^1^ HR = heart rate, BPM = beat per minutes, RT = rectal temperature. ^2^ MCS = mild–moderate cold stress. ^3^ ECS = extreme cold stress. ^a–c^ Mean values with different letters differ significantly (*p* < 0.05).

**Table 5:** The effect of cold stress on the blood cortisol, metabolites, and hematological parameters of Korean native calves in the growing stage and Korean native steers in the early fattening stage.

Items ^1^	Threshold	MCS ^2^	ECS ^3^	SEM	*p*-Value
Cortisol (ng/mL)					
calves	8.97 ^b^	9.12 ^b^	12.60 ^a^	0.894	0.014
steers	7.17 ^b^	7.00 ^b^	11.38 ^a^	0.577	<0.001
HSP70 (ng/mL)					
calves	32.30	29.64	29.17	3.067	0.741
steers	34.22	32.48	37.85	2.534	0.330
Glucose (mg/dL)					
calves	71.00 ^b^	64.50 ^ab^	61.75 ^a^	2.146	0.018
steers	64.88	68.13	70.13	2.200	0.257
NEFA (µEq/L)					
calves	118.37 ^b^	137.99 ^ab^	177.18 ^a^	16.417	0.046
steers	112.00 ^b^	113.75 ^b^	153.75 ^a^	7.948	0.002
WBC (k/µL)					
calves	11.32	11.95	10.17	0.973	0.438
steers	10.38	11.01	10.89	0.426	0.548
Lym (k/µL)					
calves	7.59	7.65	7.83	0.450	0.939
steers	7.82	8.50	8.53	0.538	0.582
Platelet (k/µL)					
calves	342.75	400.00	427.38	37.181	0.281
steers	247.75	286.63	277.38	34.575	0.712

^1^ HSP70 = heat shock protein 70, NEFA = non-esterified fatty acids, WBC = white blood cells, Lym = lymphocyte. ^2^ MCS = mild–moderate cold stress. ^3^ ECS = extreme cold stress. ^a,b^ Mean values with different letters differ significantly (*p* < 0.05).

**Table 6:** The effect of cold stress on behavioral patterns of Korean native calves in the growing stage and Korean native steers in the early fattening stage.

Items ^1^	Threshold	MCS ^1^	ECS ^2^	SEM	*p*-Value
Standing time (min/day)					
calves	251.88 ^c^	284.38 ^b^	316.25 ^a^	7.964	<0.001
steers	232.50 ^b^	257.50 ^ab^	261.88 ^a^	7.379	0.022
Lying time (min/day)					
calves	348.13 ^a^	315.63 ^b^	283.75 ^c^	7.964	<0.001
steers	367.50 ^a^	342.50 ^ab^	338.13 ^b^	7.379	0.022

^1^ MCS = mild–moderate cold stress. ^2^ ECS = extreme cold stress. ^a–c^ Mean values with different letters differ significantly (*p* < 0.05).
